# Major depressive disorder in children and adolescents is associated with reduced hair cortisol and anandamide (AEA): cross-sectional and longitudinal evidence from a large randomized clinical trial

**DOI:** 10.1038/s41398-025-03401-8

**Published:** 2025-05-24

**Authors:** Andreas Walther, Lukas Eggenberger, Rudolf Debelak, Clemens Kirschbaum, Isabelle Häberling, Ester Osuna, Michael Strumberger, Susanne Walitza, Jeannine Baumgartner, Isabelle Herter-Aeberli, Gregor Berger

**Affiliations:** 1 Clinical Psychology and Psychotherapy, University of Zurich, Zurich, Switzerland; 2 Experimental Pharmacopsychology and Psychological Addiction Research, Department of Adult Psychiatry and Psychotherapy, University Hospital of Psychiatry Zurich, University of Zurich, Zurich, Switzerland; 3 Jacobs Center for Productive Youth Development, University of Zurich, Zurich, Switzerland; 4 Psychological Methods, Evaluation and Statistics, University of Zurich, Zurich, Switzerland; 5 Biological Psychology, TU Dresden, Dresden, Germany; 6 Department of Child and Adolescent Psychiatry and Psychotherapy, Psychiatric University Hospital Zurich, University of Zurich, Zurich, Switzerland; 7 Laboratory of Human Nutrition, Institute of Food, Nutrition and Health, ETH Zürich, Zürich, Switzerland; 8 Research Department of Child and Adolescent Psychiatry, Psychiatric University Hospitals Basel, University of Basel, Basel, Switzerland; 9 Department of Nutritional Sciences, King’s College London, London, United Kingdom; 10 Laboratroy of Nutrition and Metabolic Epigenetics, Institute of Food, Nutrition and Health, ETH Zürich, Zürich, Switzerland; 11 Division of Infectious Diseases and Hospital Epidemiology, Children’s Research Centre, University Children’s Hospital Zurich, University of Zurich, Zurich, Switzerland

**Keywords:** Prognostic markers, Depression

## Abstract

Pediatric major depressive disorder (MDD) represents a leading cause of disability worldwide in children and adolescents, while its underlying pathophysiology remains largely elusive. The endocannabinoid system (ECS) and the hypothalamus-pituitary-adrenal (HPA) axis are considered intertwined regulatory systems crucially implicated in the pathophysiology of depressive disorders. This study explores the cross-sectional and longitudinal association between the ECS, specifically anandamide (AEA), and the HPA axis with its main effector cortisol and MDD status and severity in children and adolescents. Utilizing data from the omega-3-pMDD trial, a phase III Randomized Clinical Trial assessing the efficacy and safety of omega-3 fatty acid supplementation in pediatric MDD, we examined hair AEA and cortisol concentrations in 110 children and adolescents aged 8-17 years, with MDD. Associations between MDD, symptom severity and hair AEA and cortisol concentrations were explored across four measurement time points (baseline, week 6, 24 and 36). Additionally, 127 healthy children and adolescents were examined once to enable cross-sectional comparisons between MDD cases and healthy controls. Baseline comparisons for the 237 children and adolescents showed lower cortisol and AEA levels in hair of children and adolescents with MDD compared to healthy controls. Longitudinal multi-level analysis over all time-points further corroborated negative longitudinal associations between hair cortisol and depressive symptoms in children and adolescents with MDD. Taken together, reduced baseline AEA and cortisol levels emerge as robust biomarker in depressed youth, while the negative longitudinal association between hair cortisol and depression symptoms might provide useful for therapy monitoring purposes. These results hold implications for early detection, diagnosis, and therapeutic response prediction in pediatric MDD.

## Introduction

Major Depressive Disorder (MDD) represents a leading cause of disability and disease burden worldwide not only among adults, but also in children and adolescents [1–3]. Being recognized as a stress-related mental health condition, the core symptoms of MDD are persistent depressive mood and loss of interest and pleasure in all or almost all activities most of the day nearly every day [4]. Additional symptoms such as weight loss or gain, insomnia or hypersomnia, psychomotor agitation or retardation, fatigue, excessive feelings of guilt or worthlessness, decreased concentration and thoughts of death and suicide complement the clinical picture of MDD [5]. While the precise pathophysiology behind depressive disorders remain largely elusive, numerous studies have pinpointed a malfunction in the hypothalamus-pituitary-adrenal (HPA) axis, our major neuroendocrine stress response system [6, 7].

Concurrently, the endocannabinoid system (ECS) is gaining attention for its capacity to respond to and modulate stress [8]. Specifically, glucocorticoids like cortisol, pivotal to the HPA axis’s function, activate endocannabinoid signaling [8]. Therefore, the ECS and the HPA axis are considered intertwined regulatory mechanisms in the development of depressive disorders and stress-related mental issues [9–15]. This view is reinforced by the presence of glucocorticoid (GRs) and cannabinoid receptors (CBR) across key brain areas implicated in depression, such as the hypothalamus, hippocampus, cortex, and amygdala [16, 17].

Though it seemed widely accepted that MDD is associated with heightened HPA axis activity and basal cortisol levels [6, 18], recent findings, including reduced cortisol stress reactivity in MDD and inconsistent results from hair cortisol studies, challenge this view [7, 19–25].

Concerning the endocannabinoid system (ECS), research involving rodents has shown that manipulating the cannabinoid type 1 receptor (CB1R) either through genetic deletion, pharmacological inhibition, or activation enhances CB1R signaling in the brain, leading to antidepressant and anxiolytic effects [26–30]. Conversely, one study reported that blocking CB1R signaling pharmacologically was linked to antidepressant outcomes but also to heightened activity of the hypothalamic-pituitary-adrenal (HPA) axis [31]. This finding is in line with clinical studies on Rimonabant, a CB1 receptor antagonist that was initially approved for weight loss but withdrawn due to psychiatric side effects, including increased depressive symptoms, anxiety, and suicidality. Meta-analytic and clinical trial data indicate that Rimonabant significantly increased the risk of mood disorders (Christensen et al., 2007; Topol et al., 2007; Després et al., 2005). Consistent with most findings, the use of chronic unpredictable stress, a recognized model for inducing depression in rodents, has been shown to elevate HPA axis activity and decrease levels of the endocannabinoid anandamide (AEA) in the brain [32]. Similar findings emerge when giving prolonged glucocorticoid treatment to rodents [33]. Yet, in specific brain areas like the lateral habenula, increases in 2-arachidonoylglycerol (2-AG) have been observed following chronic unpredictable or social defeat stress [34], highlighting the intricate nature of these relationships when different brain regions are considered in rodent experiments. Despite these complexities, the preponderance of rodent research indicates that elevated AEA levels and the resultant enhanced CB1R signaling are linked to a decrease in depression-like symptoms [35].

Research involving human subjects, including those with MDD [9, 13], or post-traumatic stress disorder (PTSD), indicates a reduction in the serum levels of AEA and 2-AG in affected individuals [36, 37]. However, there are also findings that show no correlation, or even elevated levels, of these endocannabinoids in individuals with these conditions [38–41], as well as in those experiencing grief and loneliness, which have strong ties to MDD [42, 43]. Experimental studies have found that stress triggers the activation of fatty acid amide hydrolase (FAAH), an enzyme responsible for reducing AEA and, to a smaller extent, 2-AG levels, specifically within the amygdala [44]. Blocking FAAH, thereby boosting AEA levels, has been identified as a potential protective treatment against stress-induced anxiety in healthy subjects [45]. Another investigation revealed that prolonged stress (520 days of isolation and confinement) decreased 2-AG levels without affecting AEA in healthy males [46]. Additionally, a recent study found no link between inflammation-induced depression and circulating AEA or 2-AG levels, suggesting that changes in endocannabinoid concentrations in depressive states might not be related to inflammation [47].

Both, glucocorticoid and endocannabinoid levels in serum or the central nervous system can fluctuate based on immediate circumstances or the time of day. However, levels measured in hair, which are thought to reflect integrated long-term concentrations over several months, are generally not affected by many such variables [48]. Research has shown that individuals with borderline personality disorder have lower levels of AEA in their hair but not of 2-AG, compared to healthy individuals [49]. Additionally, there was a noted inverse relationship between depressive symptoms and hair AEA levels. Another study identified negative correlations between PTSD symptom severity and hair levels of various N-acyl-ethanolamides, including palmitoylethanolamide (PEA), oleoylethanolamide (OEA), and stearoylethanolamide (SEA) [50]. Conversely, no significant differences were found in the levels of hair 2-AG, PEA, SEA, and OEA between women currently experiencing an MDD episode and those who are not depressed [51]. A study focusing on male refugee minors found no consistent links between psychological symptoms and hair endocannabinoids, except for a positive correlation between the levels of 2-AG/1-AG and depressive symptoms [52]. Another piece of research highlighted higher levels of 1-arachidonoylglycerol (1-AG) and lower levels of SEA in women who experienced childhood maltreatment [53]. Similarly, another study suggests that greater lifetime trauma and reduced maternal hair AEA levels during pregnancy are linked to a higher risk of childbirth-related posttraumatic stress symptoms [54]. In another study involving 207 participants from the general population, negative correlations were found between hair AEA levels and symptoms of anxiety and depressive symptoms [15]. Notably, a recent study by our group revealed that hair cortisol and AEA levels were reduced in adult individuals with a positive MDD screening, while longitudinal analysis further confirmed a negative co-variation between AEA levels and depression symptoms [55]. The data stemming from adult populations further supports the view of a glucocorticoid-endocannabinoid-related stress-susceptible endophenotype in MDD as previously proposed [56, 57].

Focusing on children and adolescents, research on the relationship between MDD and hair cortisol remains until now inconclusive due to studies with small sample size and the associated risk of bias, inadequate handling of outlying and non-detectable values [58, 59], or examination of curve-linear relationships [60]. For example, no association was observed between hair cortisol and depressive symptoms in 70 children and adolescents (14 males) [61]. However, in a large cohort study of 1050 adolescents and young adults (14-21 years), for the male sample a significant association between MDD and lower baseline levels of hair cortisol was identified. This association was consistent across the entire sample over a one-year follow-up, indicating a broader trend where MDD correlates with reduced hair cortisol levels in male adolescents and young adults [62]. Why such a relationship was only identified in males requires further study. Nevertheless, smaller studies examining only male adolescents corroborate the finding of a negative association between hair cortisol and depressive symptoms [63]. Notably, in 432 adolescents (11 – 17 years old) a marginally significant negative linear association and a significant curvilinear relationship were found between hair cortisol and depressive symptoms [60]. Importantly, no data exists so far investigating AEA levels in children and adolescents with MDD.

In summary, a deficiency in hair cortisol and AEA and disruptions in CB1R signaling as well as an exhausted HPA axis seem to be implicated in adult MDD. However, to date, no investigation has been carried out aimed at examining the cross-sectional and longitudinal association between MDD status, depression severity and joint analysis of hair cortisol and AEA in children and adolescents. Based on the literature above, we postulate the following hypotheses:

1. Children and adolescents with MDD exhibit lower levels of hair cortisol and AEA compared to healthy controls.
2. In children and adolescents with MDD, hair cortisol and AEA levels are negatively associated with depressive symptoms over time.

## Methods and materials

### Study design and sample characteristics

The Omega-3-pMDD study is a completed randomized, double-blind, placebo-controlled multicenter clinical trial described in more detail elsewhere [64]. The trial consisted of a 36-week treatment phase where participants received either an omega-3 supplementation or a placebo. Ethics approval was obtained from local committees, and the trial was registered under ClinicalTrials.gov (NCT03167307).

The trial involved in total 257 depressed children and adolescents aged 8 to 17 years, all diagnosed with a MDD of moderate or high severity according to DSM-IV. Participants were recruited from inpatient and outpatient mental health services in the German-speaking region of Switzerland. Patients suffering from comorbid schizophrenia, bipolar affective disorder, substance use disorder, developmental disorders such as autism, neurological conditions or eating disorders were excluded from the trial. Other comorbid disorders, such as attention deficit disorders, anxiety disorder or conduct disorders, were not excluded when not requiring priority treatment. All patients in the trial were treated according to the S3 guidelines for depression [65]. In addition, healthy control participants for baseline comparisons were recruited and evaluated under the same conditions as the participants in the clinical trial. The healthy controls were recruited either as part of the Omega-3-pMDD clinical trial as previously described elsewhere [66] or through a companion study that specifically recruited healthy controls for comparison as previously described elsewhere (ClinicalTrials.gov: NCT04158869) [67]. Informed consent was obtained from all participants and their parents involved in the study.

For the present report, baseline measures such as the MDD diagnosis, depression severity and relevant sociodemographic variables were used, while also psychometric data from the following measurement time points at weeks 6, 24 and 36 were used. The main measure of the study was the change in scores on the Children’s Depression Rating Scale - Revised (CDRS-R) described in more detail below. Hair samples of cases were obtained at weeks 6 and 36 and further processed as described below, while healthy controls provided only baseline hair samples. For the present study, 110 depressed patients and 127 healthy control individuals provided complete psychometric and biological data for analysis. Data and code will be made available upon reasonable request.

### MDD diagnosis and children’s depression rating scale - revised (CDRS-R)

The diagnosis of MDD reflecting positive MDD status was established using the semi-structured interview Kiddie Schedule for Affective Disorders and Schizophrenia — Present and Lifetime Version (K-SADS-PL) [68]. In this study, the German version of the K-SADS-PL was used [69]. The K-SADS-PL is a diagnostic tool created to evaluate both current and past DSM-IV diagnoses in children and adolescents through interviews with the parent(s) and child. The section for diagnosing the presence of a current MDD confirmed the presence of a MDD at baseline. Additionally, the K-SADS-PL was used to assess comorbid conditions and to confirm that participants met the inclusion and exclusion criteria for the study. The K-SADS-PL shows adequate test-retest reliability and good validity [70–72].

The CDRS-R is a clinician-administered tool for assessing depression severity in children and adolescents [73–75]. This update, which includes 17 items rated on a Likert scale, aims for more precise evaluation, with the highest score indicating severe depression symptoms. This tool takes into account multiple perspectives, including that of parents, but primarily focuses on the child’s viewpoint. Poznanski et al. [75], and later Keller et al. [74], have highlighted its strong reliability and ability to distinguish between children with and without MDD, particularly noting the German version’s high internal consistency and discriminative validity.

### Hair endocannabinoid and glucocorticoid quantification

To measure hair endocannabinoids and glucocorticoids, we collected three hair samples from each subject, with each sample consisting of multiple hair strands and weighing at least 20 mg. The samples were cut as close to the scalp as possible from the back of the head. We then followed the procedures outlined in our previously published methods for analyzing glucocorticoids [76] and endocannabinoids [15]. The preprocessing and analysis of these samples were carried out at Dresden LabService GmbH in Germany. We prepared the two obtained samples of the MDD cases from trial week 6 and week 36 by cutting them first into 3 cm lengths, which reflect the hormone levels over the past three months based on an average hair growth rate of 1 cm per month [77].

Subsequently, the 3 cm segment was then halved into 1.5 cm segments to represent the 6-week period to quantify for the MDD cases the baseline period up to week 6 (segment V2 proximal) and the 6-week period prior to the baseline measurement (−6 to baseline; segment V2 distal), as well as the period between weeks 24 and 30 (segment V5 distal) and 30 and 36 (segment V5 proximal). For the healthy controls the 3 cm segment was also halved into 1.5 cm segments to represent the 6-week period prior to baseline (−6 to baseline; segment V2 proximal) and the 6-week period prior (−12 to −6; segment V2 distal). This resulted in four quantifications per person, each representing a 6-week period of hormone accumulation. Given that degradation or wash-out effects in hair samples are, if at all, typically only observed in segments longer than 3 cm. Thus, no such effects are expected in our analysis using two short 1.5 cm segments.

We weighed out 7.5 mg of hair for each test. The hair was washed with isopropanol as per Gao et al.‘s guidelines [15, 76, 78]. We employed liquid chromatography and tandem mass spectrometry (LC-MS/MS) for the biochemical analysis, as previously detailed [79]. The variability of our measurements, indicated by the intra- and inter-assay variability, was below 13% for AEA, which is within the acceptable range (less than 15% variability) according to (Caruso et al. [80]). For cortisol and cortisone, the variability was even lower, at 8.8% and 8.5% respectively. The sensitivity of our tests was high, with the lowest detectable levels being 0.003 pg/mg for AEA, 0.048 pg/mg for cortisol, and 0.194 pg/mg for cortisone. The lower LLOQ in the present study, compared to previous reports (Gao et al. [15]), are due to advances in LC-MS technology, which have substantially improved sensitivity for detecting AEA in biological matrices. However, despite this enhanced sensitivity, most AEA values in our sample were distributed at higher concentrations.

### Statistical analysis

Statistically, we checked two research questions (RQs), of which RQ1 was cross-sectional, and RQ2 was longitudinal. Each research question led to a row of statistical hypotheses, which were tested using Bayesian multilevel models. These models were estimated using the R package brms [81, 82], which allowed us to check each hypothesis. We summarize these questions and how we addressed them below:

RQ1 concerned the association between depressive status on the one hand and steroid hormones and endocannabinoids on the other hand at the time of the baseline assessment and at the measurement at six weeks. This led to the formulation of three linear regression models per time point, where a) the level of hair cortisol, b) the level of hair cortisone, and c) the level of AEA was predicted by membership to the healthy control group or the group of MDD cases. This analysis included 110 MDD cases (79 female; mean age 15.8 years) and 127 healthy controls (62 female; mean age 15.7 years) at both time points.

RQ 2 investigated the association between the raw score in CDRS-R on the one hand and steroid hormones and endocannabinoids on the other hand across the measurement timepoints of the RCT. This research question was investigated using three Bayesian multilevel models, in which the CDRS-R score was predicted by a) the level of hair cortisol, b) the level of hair cortisone, and c) the level of AEA. These models were essentially linear regression models, where the slopes and intercepts, that is, the individual regression parameters, were allowed to vary slightly for the individual participants. Allowing individual intercepts and slopes means that the model can describing how people naturally differ from one another instead of using the same prediction model for all of them. The application of Bayesian methods helps us quantify our uncertainty about these relationships quantitatively. This analysis included 110 patients (79 female; mean age 15.8 years).

For both RQ1 and RQ2, each regression model was intended to be built in three steps: 1) including only the main predictor and main outcome variable, 2) also including participants’ sex, age, and BMI as covariates, and 3) also including baseline antidepressant use as a covariate. Since we encountered convergence in the second step when building the models in RQ2, we only controlled for baseline antidepressant use as a covariate in the models of this research question.

## Results

As shown in Table 1, the study sample comprised 237 adolescents, divided into 127 healthy controls and 110 MDD cases. The average age of participants was 15.7 years (SD = 1.6) and the average BMI was 21.5 (SD = 3.8). The sample consisted of 40.5% male and 59.5% female participants. MDD cases had a higher proportion of females (71.8%) compared to healthy controls (48.8%). Regarding treatment, all MDD cases received either placebo (56.4%) or Omega-3 supplementation (43.6%), while controls did not receive any intervention. Furthermore, 41.8% of cases were on antidepressive medication at baseline. None of the controls had a current or previous psychiatric diagnosis, while 13.6% of MDD cases received a comorbid mental disorder diagnosis and 36.4% had a previous mental health disorder diagnosis that was not MDD. Supplemental Table S1 provides an overview of the bivariate Pearson correlation coefficients for all assessed biomarkers.

**Table 1:** Descriptives of the sample stratified by depression status.

	Total Sample (*n* = 237)	Healthy controls (*n* = 127)	MDD cases (*n* = 110)	*p*-value	SMD
**Treatment***, n (%)*				–	–
Placebo	–	–	62 (56.4)		
Omega-3	–	–	48 (43.6)		
**Sex***, n (%)*				**0.001**	0.484
Male	96 (40.5)	65 (51.2)	31 (28.2)		
Female	141 (59.5)	62 (48.8)	79 (71.8)		
**Age (years)***, mean (SD)*	15.7 (1.6)	15.7 (1.5)	15.8 (1.6)	0.594	0.070
**BMI***, mean (SD)*	21.5 (3.8)	21.1 (3.3)	22.0 (4.2)	0.099	0.218
**Current diagnosis**^**a**^*, n (%)*				**<0.001**	0.826
No	209 (88.2)	127 (100.0)	82 (74.5)		
Yes	15 (6.3)	0 (0.0)	15 (13.6)		
Missing	13 (5.5)	0 (0.0)	13 (11.8)		
**Previous diagnosis**^**a**^*, n (%)*				**<0.001**	1.364
No	184 (77.6)	127 (100.0)	57 (51.8)		
Yes	40 (16.9)	0 (0.0)	40 (36.4)		
Missing	13 (5.5)	0 (0.0)	13 (11.8)		
**Antidepressive medication at baseline***, n (%)*				**<0.001**	1.199
No	191 (80.6)	127 (100.0)	64 (58.2)		
Yes	46 (19.4)	0 (0.0)	46 (41.8)		
**Baseline questionnaire scores,** *mean (SD)*					
CDRS-R	40.5 (20.4)	19.3 (3.0)	57.9 (8.6)	**<0.001**	5.998
**Baseline hair concentrations,** *mean (SD)*					
Cortisol (pg/mg)	3.5 (3.5)	4.1 (4.1)	2.7 (2.4)	**0.001**	0.420
Cortisone (pg/mg)	7.1 (6.1)	7.0 (7.0)	7.2 (4.9)	0.774	0.037
AEA (pg/mg)	2.4 (2.8)	3.0 (3.0)	1.7 (2.3)	**<0.001**	0.485

*MDD* major depressive disorder, *n* number of participants, *SD* standard deviation, *SMD* standardized mean difference.

^a^any kind of psychiatric diagnosis, except MDD.

Bold p-values mark significant differences between healthy controls and MDD cases.

**Fig. 1:**
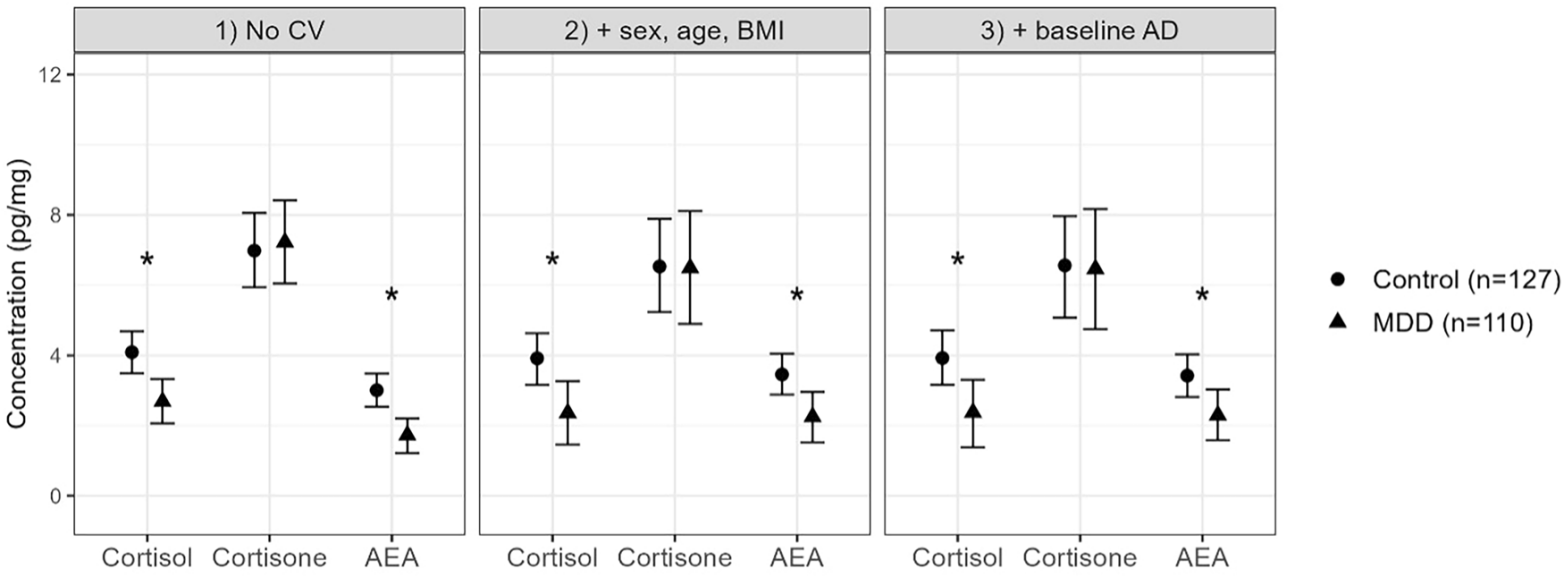
Conditional effects of depressive symptoms (CDRS-R scores) on Cortisol, Cortisone and AEA at baseline. Left panel shows analysis without covariates (CV), center panel shows analysis with sociodemographic covariates (age, sex and BMI), and right panel shows analysis with sociodemographic covariates and baseline antidepressant (AD) medication (*n* = 237). Asterisks indicate statistically significant differences between control and MDD group using a 95% credible interval.

Regarding the association of depressive status with steroid hormones and endocannabinoids at baseline and week six (RQ1), depressive status at baseline measurement was negatively associated with cortisol (Median = −1.41, CI_95_ = [−2.29; −0.55]) and AEA (Median = −1.20, CI_95_ = [−1.96; −0.64]), but not with cortisone (Median = 0.23, CI_95_ = [−1.38; 1.83]) levels. Controlling for participants’ age, sex, and BMI, as well as additionally controlling baseline antidepressant use did not change statistical significance of these associations (Fig. 1). At the six-week measurement, the same associations were observed as at the baseline measurement, with one exception: depressive status and cortisol showed no bivariate association (Median = −0.85, CI95 = [−1.74, 0.01]). However, this association became significant when covariates were introduced into the model (Median = −1.32, CI95 = [−2.36, −0.28]).

Additional sensitivity analyses for other endocannabinoids are presented in the Supplement (Table S2, Table S3). Additional sensitivity analysis examining a potential modulating effect of antidepressant medication on AEA levels is presented in the supplement (Text S1, Figure S1). Furthermore, additional sensitivity analyses examining potential sex differences in the association between MDD status and cortisol and AEA are presented in the supplement (Text S2, Figure S2).

As for the association of the CDRS-R with steroid hormones and endocannabinoids across measurement timepoints (RQ2), cortisol (Median = −0.69, CI_95_ = [−1.17; −0.22]; SD = 0.40, CI_95_ = [0.02; 1.10]) and cortisone levels (Median = −0.30, CI_95_ = [−0.53; −0.06]; SD = 0.27, CI_95_ = [0.01; 0.70]) were negatively associated with the CDRS-R score reflecting depression severity across measurement timepoints. No association was found for AEA levels (Median = 0.13, CI_95_ = [−0.84; 1.07]; SD = 1.30, CI_95_ = [0.11; 3.05]) with the CDRS-R score. Models where we controlled for participants’ age, sex, and BMI showed numerical convergence problems and are therefore not reported. When we controlled for baseline antidepressant use, we still found a negative association for cortisol (Median = −0.71, CI_95_ = [−1.19; −0.24]; SD = 0.43, CI_95_ = [0.02; 1.14]) and cortisone (Median = −0.29, CI_95_ = [−0.53; −0.06]; SD = 0.27, CI_95_ = [0.01; 0.67]) levels with the CDRS-R score. There was no association found for AEA levels with the CDRS-R score (Median = 0.13, CI_95_ = [−0.84; 1.07]; SD = 1.30, CI_95_ = [0.09; 3.01]).

No significant relationships were found when predicting X1AG-2AG levels (Median = 0.30, CI_95_ = [−0.27; 2.11]; SD = 3.56, CI_95_ = [0.76; 2.11]), OEA levels (Median = −54.90, CI_95_ = [−132.01; 18.37]; SD = 111.02, CI_95_ = [12.58; 220.67]), PEA levels (Median = −69.57, CI_95_ = [−177.45; 34.99]; SD = 207.07, CI_95_ = [29.63; 353.50]) and SEA levels (Median = −7.69, CI_95_ = [−32.00; 15.97]; SD = 71.70, CI_95_ = [46.14; 99.13]) from cortisol levels while controlling for baseline depression. Similar results were found when predicting X1AG-2AG levels (Median = 0.55, CI_95_ = [0.09; 1.04]; SD = 0.90, CI_95_ = [0.05; 1.92]), OEA levels (Median = −32.36, CI_95_ = [−66.22; 1.27]; SD = 27.69, CI_95_ = [1.10, 73.10]), PEA levels (Median = −17.71, CI_95_ = [−71.47; 34.51]; SD = 99.57, CI_95_ = [37.03; 165.33]) and SEA levels (Median = −8.50, CI_95_ = [−18.38; 1.01]; SD = 17.57, CI_95_ = [3.01; 29.58]) from cortisone levels while controlling for baseline depression, with only X1AG-2AG levels showing a positive association. No significant relationships were found when predicting X1AG-2AG levels (Median = 2.53, CI_95_ = [0.46; 4.80]; SD = 5.89, CI_95_ = [3.21; 8.80]), OEA levels (Median = −38.71, CI_95_ = [−200.08; 106.51]; SD = 286.57, CI_95_ = [38.94, 541.99]), PEA levels (Median = −70.24, CI_95_ = [−353.10; 132.33]; SD = 419.44, CI_95_ = [51.01; 1043.27]) and SEA levels (Median = −66.14, CI_95_ = [−340.22; 131.16]; SD = 410.12, CI_95_ = [52.35; 1040.05]) from AEA levels while controlling for baseline depression.

**Fig. 2:**
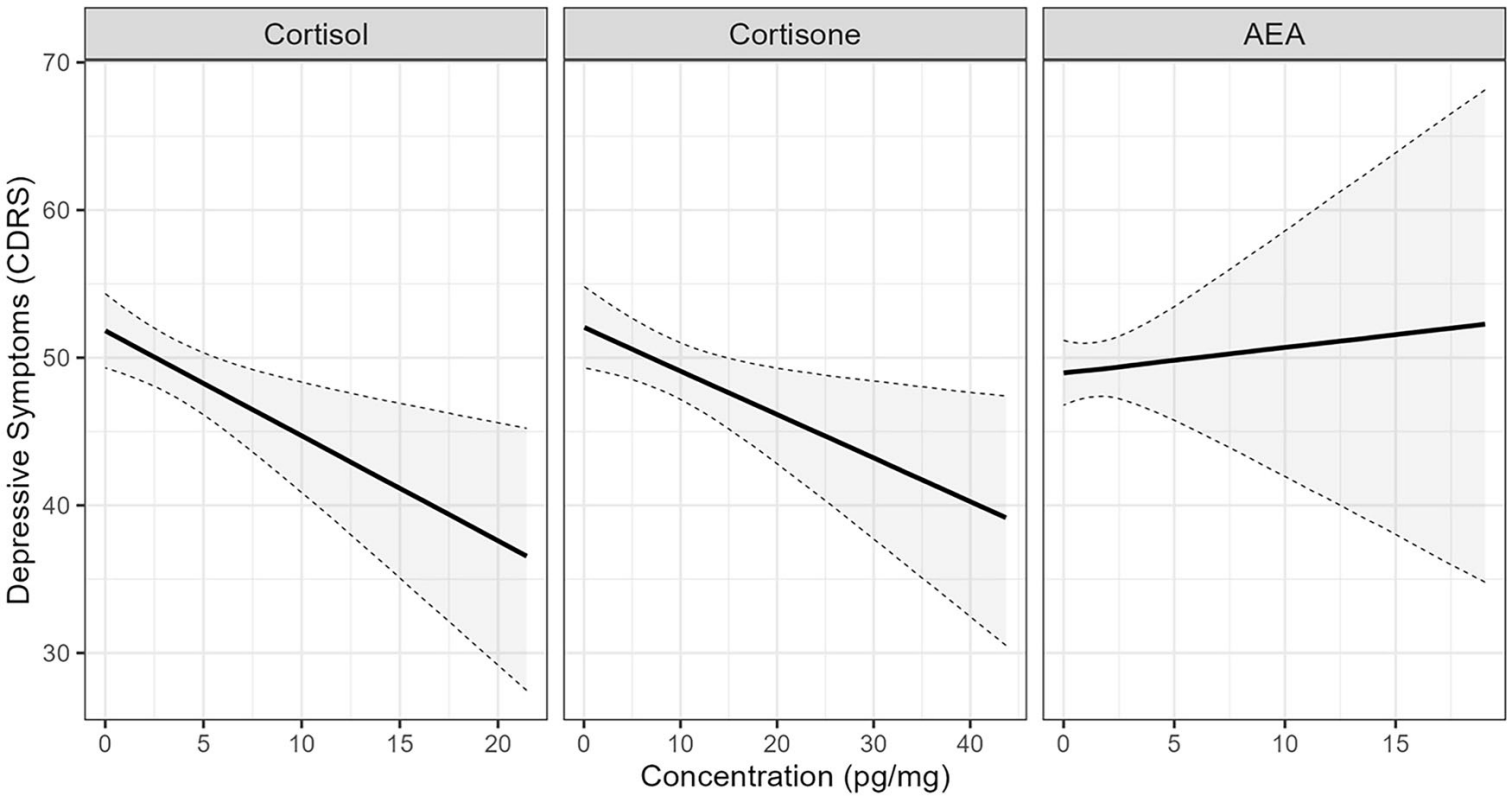
Conditional effect of Bayesian multilevel modeling for depressive symptoms (CDRS-R scores) being predicted by Cortisol, Cortisone and AEA across all time points for MDD cases (n = 110), controlled for baseline antidepressant medication.

Figure 2 depicts the conditional effect of cortisol, cortisone and AEA on the CDRS-R. Additional analysis not shown in the manuscript showed no effect of Omega-3 supplementation on cortisol, cortisone or AEA levels across the four time points in MDD cases.

## Discussion

The findings of the present study concerning reduced baseline levels of hair cortisol and AEA and a consistent negative relationship between hair cortisol and depressive symptoms in children and adolescents suffering from MDD add to the narrative of neuroendocrine and endocannabinoid involvement in depressive disorders. These findings corroborate and expand previous research underscoring a dysregulation in the HPA axis and the ECS as pivotal elements in the pathophysiology of depression, albeit with a novel focus on pediatric populations.

### Reduced hair cortisol in pediatric MDD

The observed reduction in hair cortisol levels among children and adolescents with MDD compared to healthy controls in the study parallels the established notion of HPA axis dysregulation in depressive disorders. However, it contradicts the traditional view that depression is primarily associated with heightened cortisol levels due to chronic stress activation of the HPA axis [18]. This discrepancy invites a reevaluation of the biological mechanisms at play, especially in pediatric populations.

One plausible explanation for this phenomenon could involve an adaptive downregulation of the HPA axis in response to prolonged stress, leading to reduced cortisol production over time. This theory is supported by evidence suggesting that chronic stress can lead to glucocorticoid receptor resistance, thereby blunting the HPA axis response and reducing cortisol output [7, 83–85]. Additionally, genetic factors may influence cortisol secretion and stress sensitivity, contributing to the variability in HPA axis activity observed in MDD [86, 87].

However, it is also plausible, that the HPA axis might be already hypoactive from the outset and does not function as it should. This could therefore mean that cortisol secretion is generally too low in children with MDD and that the entire HPA axis simply secretes insufficient cortisol in its basic activity. Several studies investigating hair cortisol in depressed individuals support this notion [7, 22, 25, 88].

There are also findings, that adults with MDD show too little secretion in the Trier Social Stress Test in response to psychosocial stress [20]. However, it must be noted that this is primarily true for women, which in turn would be consistent with the present sample, as there is an excess of girls in the examined MDD cases. Furthermore, the conversion rate of cortisol to cortisone might be too high, so that all the active cortisol is converted into biologically inactive cortisone – this perspective is supported by the fact that cortisone was not reduced despite the lower cortisol levels. However, the fact that only cortisol is lowered and not cortisone could indicate that the enzyme 11-B-HSD, which converts cortisol into cortisone is overactive and inactivates the important energy-providing active hormone cortisol systemically too quickly, leaving the system with too limited energy to remain active and respond to stress.

The finding, that hair cortisol is longitudinally negatively associated with depressive symptoms in children and adolescence with MDD indicates that once a MDD is established the HPA axis is exhausted and does not recover from the ongoing condition. This suggests that pharmacological treatment with synthetic glucocorticoids should be investigated in this population. This is despite the fact that it has long been assumed that antiglucocorticoid therapies are more likely to be indicated for MDD [89].

### Reduced hair AEA in pediatric MDD

The negative cross-sectional association between AEA levels and depression severity in pediatric populations resonates with the broader literature emphasizing the critical role of the ECS in emotional regulation and stress response [8]. AEA, known for its neuroprotective and mood-enhancing effects, might be depleted in pediatric MDD, potentially reflecting an impaired stress coping mechanism or a dysregulated ECS [13]. This depletion of AEA in MDD could result from increased activity of enzymes like fatty acid amide hydrolase (FAAH), which metabolizes AEA, leading to lower availability of this mood-regulating endocannabinoid [57]. The reduced AEA levels could also be a consequence of altered cannabinoid receptor (CB1) function or expression, further disrupting the endocannabinoid signaling crucial for emotional equilibrium and stress resilience.

Intriguingly, while the current study and a previous study conducted in adults [55], both identified baseline cross-sectional differences for hair cortisol and AEA in their respective populations, the longitudinal associations diverged. In the study involving adults, depressive symptoms were negatively associated with hair AEA levels over time, but not with hair cortisol, suggesting a potential age-related difference in the neurobiological underpinnings of MDD and its progression. This divergence underscores the complexity of the HPA axis and ECS interplay in depressive disorders and highlights the need for age-specific research to unravel the nuanced mechanisms governing these associations.

Moreover, the finding that controlling for baseline antidepressant use results in a continued significant association between depressive status and lower hair cortisol levels, while the association with hair AEA is no longer significant, provides an intriguing insight into the differential regulation of these biomarkers in pediatric major depressive disorder (MDD). This suggests that while cortisol levels may be inherently linked to the pathophysiology of depression, AEA levels might be more sensitive to external influences, such as medication, which could mitigate the observed differences. This distinction emphasizes the robust role of the HPA axis in depression, potentially independent of immediate pharmacological interventions, whereas the ECS may exhibit a more dynamic response to treatment [10, 57].

### Limitations

When interpreting the results, several limitations must be considered. First, the inclusion of participants with comorbid conditions such as ADHD, anxiety disorders, and behavioral disorders could have confounded the results. These comorbidities may introduce additional variability, potentially biasing the outcomes and complicating the interpretation of the relationship between depression and endocannabinoid levels. Second, some of the study participants had undergone psychotherapy or medication treatments also before the start of the study. These prior interventions could affect the levels of hair glucocorticoids and endocannabinoids thereby influencing the study’s findings. Third, the study, although for this specific population being relatively large, was not large enough to include a wide range of confounding variables, such as the use of hormonal contraceptives, particularly relevant for female participants. Given the potential impact of hormonal changes on endocannabinoid levels, the omission of this variable could skew results and limit the applicability of the findings to broader populations. Furthermore, due to the multicenter structure of the trial and the vulnerable nature of the pediatric MDD population, extended sociodemographic variables such as race/ethnicity or socioeconomic status were not systematically collected. This limits the ability to explore potential moderating effects of these factors and may constrain the generalizability of the findings to more diverse populations. Fourth, the participants had an average age of 15.7 years with a standard deviation of 1.6. This narrow age range means the findings may not be applicable to younger, prepubertal children or to adults, restricting the generalizability of the conclusions. Fifth, the findings from this pediatric sample may not directly translate to adult populations. Different physiological, emotional, and social dynamics in adults could lead to different interactions between depression and endocannabinoid levels, necessitating further research in diverse age groups. These limitations underscore the need for additional studies, particularly those that address these gaps, to better understand the complex dynamics between depression and endocannabinoid systems across different populations and life stages.

## Conclusion

The present findings regarding reduced hair cortisol and AEA levels in pediatric MDD not only corroborate the existing literature on the HPA axis and ECS dysregulation in MDD but also spotlight the unique aspects of these biomarkers in a younger population. The contrasting longitudinal findings between pediatric and adult populations call for further research into the developmental trajectories of these systems and their interaction in the context of depressive disorders. Understanding these complex dynamics will be crucial for developing targeted interventions and personalized treatment strategies for MDD across the lifespan. These results hold implications for early detection, diagnosis, and therapeutic response prediction in pediatric MDD. However, further research is required to corroborate these findings and to deepen our understanding of the HPA axis, the ECS and their interplay in pediatric MDD.

## Supplementary information

Supplementary

## Data Availability

Data will be made available upon reasonable request.

## Code availability

Code will be made available upon reasonable request.
