# Supplementary material for: Major depressive disorder in children and adolescents is associated with reduced hair cortisol and anandamide (AEA): cross-sectional and longitudinal evidence from a large randomized clinical trial
Source: Transl Psychiatry. 2025 May 24;15:183. doi: 10.1038/s41398-025-03401-8 (PMC12103555; doi:10.1038/s41398-025-03401-8)

Major Depressive Disorder in Children and Adolescents is Associated with Reduced Hair Cortisol and Anandamide (AEA): Cross-sectional and Longitudinal Evidence from a Large Randomized Clinical Trial

Walther, A.[1,*]; Eggenberger, L.[2,3]; Debelak, R.[4] Kirschbaum, C.[5]; Häberling, I.[6]; Osuna, E.[7]; Strumberger, M.[8]; Walitza, S.[6]; Baumgartner, J.[9,]; Herter-Aeberli, I.[10]; Berger, G.[6]

[1] Clinical Psychology and Psychotherapy, University of Zurich, Zurich, Switzerland
[2] Experimental and Clinical Pharmacopsychology, Department of Adult Psychiatry and Psychotherapy, University Hospital of Psychiatry Zurich, University of Zurich, Zurich, Switzerland
[3] Jacobs Center for Productive Youth Development, University of Zurich, Zurich, Switzerland
[4] Psychological Methods, Evaluation and Statistics, University of Zurich, Zurich, Switzerland
[5] Biological Psychology, TU Dresden, Dresden, Germany
[6] Department of Child and Adolescent Psychiatry and Psychotherapy, Psychiatric University Hospital Zurich, University of Zurich, Zurich, Switzerland
[7] Laboratory of Human Nutrition, Institute of Food, Nutrition and Health, ETH Zürich, Switzerland
[8] Research Department of Child and Adolescent Psychiatry, Psychiatric University Hospitals Basel, University of Basel, Basel, Switzerland
[9] Department of Nutritional Sciences, King's College London, London, United Kingdom
[10] Laboratroy of Nutrition and Metabolic Epigenetics, Institute of Food, Nutrition and Health, ETH Zürich, Switzerland

---

Supplementary

Date: 4th of January, 2025

Correspondence to: Dr. Andreas Walther, Department of Clinical Psychology and Psychotherapy, Psychological Institute, University of Zurich, Zurich, Switzerland. Email: a.walther@psychologie.uzh.ch

Table S1
Bivariate Pearson Correlation Matrix for Baseline Biomarkers (*n* = 237)

	Cortisol	Cortisone	AEA	1AG-2AG	OEA	PEA	SEA
Cortisol	–						
Cortisone	**.42*****	–					
AEA	.09	-.12	–				
1AG-2AG	.10	-.05	**.29*****	–			
OEA	.04	-.11	.13	**.29*****	–		
PEA	-.05	-.07	-.01	.14	**.65*****	–	
SEA	-.02	-.17	**.46*****	**.28*****	**.53*****	**.62*****	–

Note. *p*-values were adjusted for multiple testing using the Holm-method.
*** $p < .001$

Table S2
Effect of Depressive Status on Other Endocannabinoid Concentrations at Baseline Measurement (*n* = 237)

	**1) No CV**		**2) + age, sex, bmi**		**3) + baseline AD**	
Outcome	Estimate	95% CI	Estimate	95% CI	Estimate	95% CI
1AG-2AG	-1.3	[-9.0; 6.3]	-2.4	[-10.2; 5.5]	-2.2	[-11.5; 6.9]
OEA	417.1	[-143.5; 967.0]	384.1	[-203.3; 984.8]	407.4	[-276.9; 1087.6]
PEA	1221.1	[580.2; 1835.0]	1164.1	[522.2; 1805.9]	1360.3	[616.8; 2095.3]
SEA	-4.1	[-149.6; 144.5]	-10.0	[-160.0; 138.3]	77.1	[-95.6; 246.0]

Note. CV = covariates; AD = antidepressant medication; CI = credible interval.

Table S3
Effect of Cortisol on Other Endocannabinoid Concentrations Across Measurements (*n* = 237)

	**1) No CV**		**2) + age, sex, BMI**		**3) + baseline AD**	
Outcome	Estimate	95% CI	Estimate	95% CI	Estimate	95% CI
1AG-2AG	0.91	[-0.13; 2.01]	-	-	0.88	[-0.27; 2.11]
OEA	-44.22	[-115.00; 25.62]	-	-	-54.90	[-132.01; 18.37]
PEA	-57.58	[-156.98; 38.60]	-	-	-69.57	[-177.45; 34.99]
SEA	-5.89	[-28.05; 15.79]	-	-	-7.69	[-32.00; 15.97]

Note. CV = covariates; AD = antidepressant medication; CI = credible interval.

Table S4
Effect of Cortisone on Other Endocannabinoid Concentrations Across Measurements (*n* = 237)

	**1) No CV**		**2) + age, sex, BMI**		**3) + baseline AD**	
Outcome	Estimate	95% CI	Estimate	95% CI	Estimate	95% CI
1AG-2AG	0.60	[0.19; 1.06]	-	-	0.55	[0.09; 1.04]
OEA	-27.74	[-58.11; 2.49]	-	-	-32.36	[-66.22; 1.27]
PEA	-13.49	[-63.34; 35.00]	-	-	-17.71	[-71.47; 34.51]
SEA	-7.25	[-16.39; 1.53]	-	-	-8.50	[-18.38; 1.01]

Note. CV = covariates; AD = antidepressant medication; CI = credible interval.

Table S5
Effect of AEA Level on Other Endocannabinoid Concentrations Across Measurements (*n* = 237)

	**1) No CV**		**2) + age, sex, BMI**		**3) + baseline AD**	
Outcome	Estimate	95% CI	Estimate	95% CI	Estimate	95% CI
1AG-2AG	2.69	[0.70; 4.83]	-	-	2.53	[0.46; 4.80]
OEA	-51.57	[-210.26; 91.42]	-	-	-38.71	[-200.08; 106.51]
PEA	-70.77	[-332.85; 127.50]	-	-	-70.24	[-353.10; 132.33]
SEA	-72.52	[-345.26; 128.15]	-	-	-66.14	[-340.22; 131.16]

Note. CV = covariates; AD = antidepressant medication; CI = credible interval.

Text S1

As detailed in Figure S1, analysis of AEA concentrations among the three groups (control, MDD with antidepressants, and MDD without antidepressants) revealed lower AEA concentrations in both MDD groups, regardless of antidepressant use. However, no difference was found between MDD children with and without antidepressants.

Figure S1

Tukey-Posthoc Analysis of AEA Concentrations Stratified by Depressive Status and Antidepressant Medication Use at Baseline

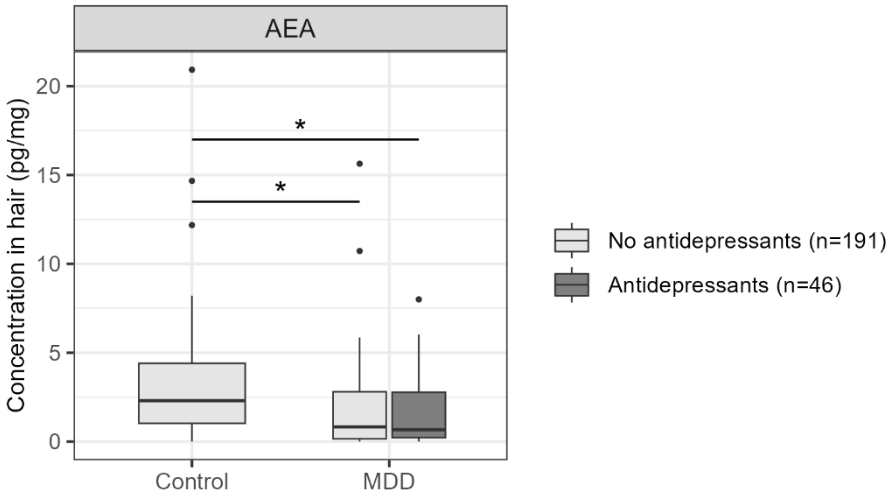

Text S2

We conducted two-way ANOVA to analyze the association of sex, depressive status, and their potential interaction on cortisol and AEA levels. The cortisol model showed only a main effect of depressive status on cortisol levels [$F(1,230) = 9.97$, $p = .002$, $\eta^2 = .04$], but no sex effect [$F(1,230) = 0.77$, $p = .381$, $\eta^2 = <.01$] and no interaction effect [$F(1,230) = 0.57$, $p = .449$, $\eta^2 = .04$].

The AEA model showed significant main effects for both depressive status [$F(1,230) = 14.62$, $p <.001$, $\eta^2 = .06$] and sex [$F(1,230) = 5.29$, $p = .022$, $\eta^2 = .02$], as well as a significant interaction effect of depressive status and sex [$F(1,230) = 7.52$, $p = .001$, η2 = .03]. Tukey-post-hoc tests showed that male children in the control group had significantly higher AEA levels compared to all other groups (Figure S2).

Figure S2

Tukey-Posthoc Analysis of Cortisol and AEA Concentrations Stratified by Depressive Status and Sex at Baseline

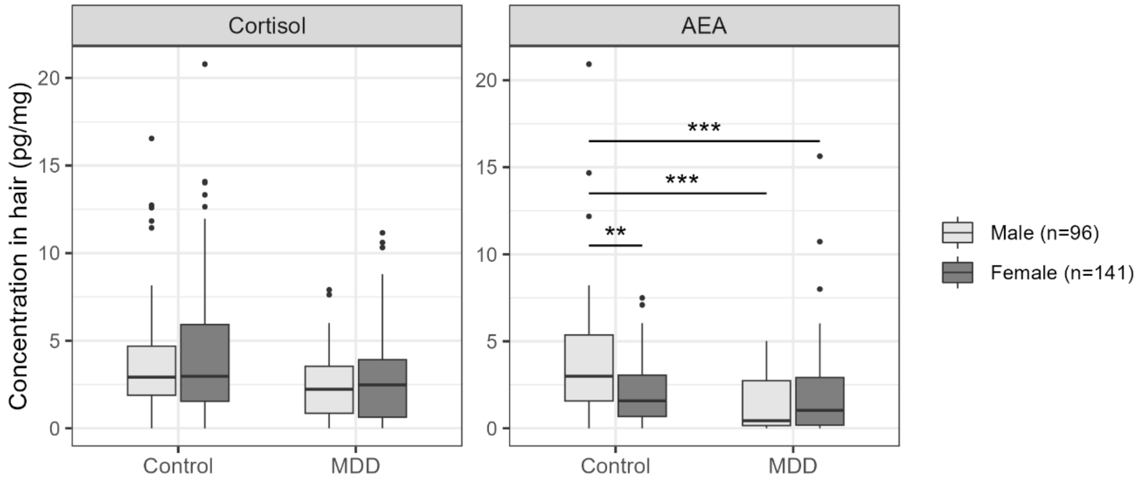

Supplement: Supplementary file 1 — Supplementary [file 41398_2025_3401_MOESM1_ESM.pdf]
